# Rolling Bearing Fault Diagnosis Based on a Synchrosqueezing Wavelet Transform and a Transfer Residual Convolutional Neural Network

**DOI:** 10.3390/s25020325

**Published:** 2025-01-08

**Authors:** Zihao Zhai, Liyan Luo, Yuhan Chen, Xiaoguo Zhang

**Affiliations:** 1 School of Information and Communication, Guilin University of Electronic Technology, Guilin 541004, China; zihaozhai@mails.guet.edu.cn (Z.Z.); xiaoguozhang@mails.guet.edu.cn (X.Z.); 2 School of Computer Science and Information Security, Guilin University of Electronic Technology, Guilin 541004, China; 2200520401@mails.guet.edu.cn

**Keywords:** fault diagnosis, synchrosqueezing wavelet transform (SWT), transfer learning, transfer residual convolutional neural network (TRCNN)

## Abstract

This study proposes a novel rolling bearing fault diagnosis technique based on a synchrosqueezing wavelet transform (SWT) and a transfer residual convolutional neural network (TRCNN) designed to address the difficulties of feature extraction caused by the non-stationarity of fault signals, as well as the issue of low fault diagnosis accuracy resulting from small sample quantities. This approach transforms the one-dimensional vibration signal into time–frequency diagrams using an SWT based on complex Morlet wavelet basis functions, which redistributes (squeezes) the values of the wavelet coefficients at different localized points in a time–frequency plane to the estimated instantaneous frequencies. This allows the energy to be more fully concentrated in actual corresponding frequency components. This strategy improves both the time–frequency aggregation and the resolution, which better reflects the eigenvalues of non-stationary signals. In this process, transfer learning and a residual structure are used in the training of a convolutional neural network. The resulting time–frequency diagrams, acquired using the steps discussed above, are then input to the TRCNN for diagnosis. A series of validation experiments confirmed that applying the TRCNN structure made it possible to achieve high diagnostic accuracy, even when training the network with only a small number of fault samples, as all 12 fault types from the test dataset were diagnosed correctly. Further simulation experiments demonstrated that our proposed method improved fault diagnosis accuracy compared to that of conventional techniques (with increases of 1.74% over RCNN, 1.28% over TCNN, 1.62% over STFT, 1.73% over WT, 2.83% over PWVD, and 1.39% over STFA-PD). In addition, diagnostic accuracy reached 100% during the application of three-time transfer learning, validating the effectiveness of the proposed method for rolling bearing fault diagnosis.

## 1. Introduction

Rolling bearing fault diagnosis has been applied in a variety of fields, including industrial manufacturing and the energy industry. By monitoring vibration signals from the bearings and other system components, signs of failure can be detected at an early stage to prevent mechanical issues and extend equipment service time, which reduces maintenance costs and improves productivity.

In practical industrial production, current problems in the field of rolling bearing fault diagnosis include problems with difficult eigenvalue extraction caused by the non-stationarity of fault signals and problems with low fault diagnostic accuracy due to limited sample sizes.

The non-stationarity of rolling bearing fault signals leads to significant difficulty in extracting fault eigenvalues. First, fault signals contain impact signals caused by the rolling body, inner ring, outer ring, and other faulty parts, which are transient and non-periodic. As such, these signals may be affected by the bearing speed, load, and other dynamic factors in the mechanical system, implying spectral components in the signal change over time, and the spectral characteristics are not fixed. Second, the intensity and location of the fault impact components in the time domain signal vary with time, while conventional frequency domain analysis assumes that the signal is smooth (i.e., frequency components do not vary with time throughout the analysis window), which complicates the acquisition of effective feature information using a traditional fixed-window length or linear analysis method. In addition, during the operation of rolling bearings, fault signals are heavily masked by intense mechanical noise and other structural vibration signals. The impact components of fault signals are dispersed in the time–frequency domain, making it difficult for traditional noise reduction methods (such as band-pass filtering or low-pass filtering) to effectively isolate the fault signals.

Small numbers of fault samples lead to low fault diagnosis accuracy. Multiple failure modes exist for rolling bearings, and the fault types can be categorized into outer ring faults, inner ring faults, and rolling body faults. There are also various fault sizes and loads, and fault characteristics are often obscured by high-noise backgrounds. Thus, when the number of samples is insufficient, the model struggles to learn comprehensive fault patterns during training, resulting in poor generalization ability under varying conditions. Additionally, deep learning models require large numbers of samples to learn complex features. When the sample size is small, deep learning models are prone to overfitting and unstable gradient updates, which affects model convergence and thus, reduces diagnostic accuracy.

During routine operation, changes in load, speed, and other factors can introduce non-stationary and non-linear vibrations into rolling bearings [1], which complicates the extraction process [2,3,4]. As such, joint distribution information in both the time and frequency domains is needed to accurately characterize the local features of non-stationary signals [5,6,7]. The wavelet transform (WT) is a typical joint time–frequency analysis technique which can be used to analyze signals at different scales. By selecting an appropriate wavelet basis and coefficient threshold, rolling bearing fault signals that are potentially non-stationary or contain transient impulse terms can be analyzed in a targeted manner, which effectively removes noise and improves the recognizability of the fault characteristics. For example, Wei et al. [8] proposed a rolling bearing fault diagnosis model that used a continuous wavelet transform (CWT) and residual networks based on an extreme learning machine. Tao [9] investigated a bearing fault diagnosis method that combined wavelet transforms and a support vector machine (SVM). First, the bearing vibration signal was processed using wavelet analysis to extract features, and the SVM was then employed for classification. Experimental results showed that the accuracy of fault diagnosis using this method exceeded 90%.

The introduction of machine learning (ML) has accelerated fault diagnosis research, providing several new methodologies for processing and analyzing collected data. Li et al. were among the first to use neural networks for this purpose, implementing a time and frequency-domain bearing vibration analysis [10]. Similarly, Shao et al. applied an optimization deep belief network specifically to rolling bearing fault prediction [11]. Other techniques have involved the use of attention mechanisms [12], adaptive neural networks [13,14,15], fuzzy models [16], support vector machines [17], and deep learning architectures [18,19,20,21]. Convolutional neural networks (CNNs) are common deep learning models used for fault diagnosis [21,22,23,24]. In this framework, a multi-layer nonlinear structure is used to automatically extract device-state fault features directly from the original input signal, thereby overcoming the limitations of conventional shallow classifiers that have difficulty capturing complex nonlinear relationships [25,26,27,28]. Wang et al. [29] combined a channel attention mechanism from a squeeze excitation network with a CNN and proposed a bearing fault diagnosis method based on a symmetrical point map and a squeeze excitation convolutional neural network (SE-CNN), which exhibited good stability and generalizability. Wang et al. [30] significantly improved the accuracy of fault diagnosis by extracting features from the original vibration signal and an acoustic signal, employing a 1D CNN for data fusion.

Similarly, transfer learning (TL) uses knowledge gained from a source domain to assist in solving learning tasks in the target domain. Compared with building a model from scratch, this method of transferring a pre-trained model to the target task can typically achieve higher learning efficiency with a smaller sample size, thereby speeding up convergence. This approach also overcomes problems encountered by CNNs in rolling bearing fault diagnosis, including poor learning capabilities with a small number of samples, low diagnostic accuracy, and the need for large quantities of resources to make network parameter adjustments. As such, the ability to achieve accurate fault diagnosis with only a small number of fault samples is a significant advantage [31]. Li et al. [32] transferred fault diagnosis knowledge learned from existing data in the source domain to the target domain, demonstrating the effectiveness of transfer learning for solving problems in similar domains using simulation experiments. Chen et al. [33] proposed a fault diagnosis method based on transfer learning with deep neural networks. By transferring certain network parameters to the target task, this method could effectively detect early bearing faults in cases with small sample quantities, offering a distinct advantage over traditional methods.

To address the difficulty of eigenvalue extraction (complicated by the non-stationarity of signals) and the low accuracy of diagnosis (characteristic of small sample sizes), we propose a rolling bearing fault diagnosis method that combines a synchrosqueezing wavelet transform (SWT) and a transfer residual convolutional neural network (TRCNN). This redistributes the wavelet coefficients at different local points on the time–frequency plane and converts the time-domain vibration signal into time–frequency diagrams, effectively revealing the features of non-stationary signals. These time–frequency diagrams, which exhibit the characteristics of high time–frequency aggregation and resolution, can then be input to the TRCNN for training. The use of transfer learning and a residual structure overcomes the problem of low diagnostic accuracy, which often occurs when training with a limited number of samples. As a result, 12 fault types were diagnosed with 100% accuracy after the application of three-time transfer learning.

The remainder of this paper is organized as follows. Section 2 describes the improved algorithm based on the SWT and TRCNN. Section 3 introduces the experimental analysis and validation steps using the Case Western Reserve University (CWRU) bearing dataset. Section 4 provides comparisons between the proposed method and several existing methods. Section 5 summarizes the paper and draws conclusions.

## 2. Proposed Method

### 2.1. Rolling Bearing Fault Diagnosis Model

**Figure 1:**
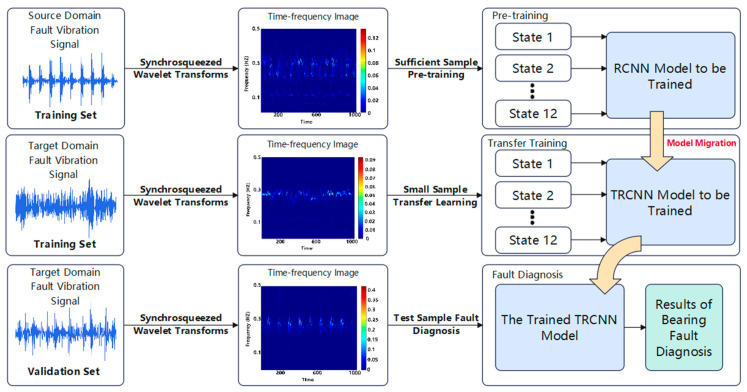
The algorithm architecture.

The proposed rolling bearing fault diagnosis method, which combines an SWT and a TRCNN, is designed to address the difficulty of extracting eigenvalues from non-stationary signals and the low diagnostic accuracy caused by small sample sizes. The model architecture is shown in Figure 1, and the corresponding methodology can be described as follows.

Step 1: Obtain rolling bearing vibration signals under different working conditions and transform these signals from the time domain into time–frequency diagrams in the time–frequency domain using an SWT.

Step 2: Pre-train the source domain data using a residual convolutional neural network (RCNN) model.

Step 3: Retain the structure and weights of the pre-trained RCNN and transfer them to the target domain. Set the weight attributes for all model layers as trainable and construct the TRCNN model.

Step 4: Fine-tune the TRCNN using a small learning rate with the dataset in the target domain.

Step 5: Evaluate the model using time–frequency diagrams acquired from the test dataset, intended for the diagnosis of bearing faults under different operating conditions.

### 2.2. Synchrosqueezed Wavelet Transform

Synchrosqueezed wavelet transform (SWT) is a type of signal processing technique that offers good adaptability for the processing of non-stationary and non-linear signals [34], which are commonly observed in fault vibration data.

In this study, rolling bearing vibration signals, represented as y=x(t), are processed using an SWT during time–frequency analysis. The complex Morlet wavelet basis function, composed of a complex exponential multiplied by a Gaussian window, was selected for the SWT. This wavelet is highly sensitive to sharp discontinuities and transient shocks in non-stationary and nonlinear fault signals. As such, it is able to efficiently capture frequency changes associated with bearing faults, allowing for the accurate identification of fault characteristics and background noise [35].

In this process, a synchrosqueezed wavelet transform was applied to the vibration signal. The set of complex Morlet wavelet basis functions could be represented as $\phi (t)[\phi (t)\in L^{2}(R)]$. The continuous wavelet function could then be obtained by stretching and translating this term, yielding the following [34]:

(1) $$\phi_{a,b}\left(t\right)=\frac{1}{\sqrt{a}}\phi \left(\frac{t-b}{a}\right),a>0,b\in R,$$

where *a* is a stretch factor, and *b* is a translation factor. The continuous wavelet transform of $x(t)\in L^{2}(R)$ is then given by the following [34]:

(2) $$CWT_{x}\left(a,b\right)=\int_{-\infty }^{+\infty }x\left(t\right){\bar{\phi }}_{a,b}\left(t\right)dt=\int_{-\infty }^{+\infty }x\left(t\right)\sqrt{a}\phi \left(\frac{\bar{t-b}}{a}\right)dt,$$

where $CWT_{x}(a,b)$ represents wavelet coefficients. Next, a synchronized squeezing processing is performed on the wavelet coefficients. With $\omega_{l}$ as the center frequency and $\left[\omega_{l}-\Delta \omega /2,\omega_{l}+\Delta \omega /2\right]$ as the frequency band, the synchrosqueezed wavelet transform at discrete scale $a_{k}$ is obtained as follows [34]:

(3) $$T_{x}(\omega_{l},b)=(\Delta \omega {)}^{-1}\sum_{a_{k}:\left|\omega \left(a_{k},b\right)-\omega_{l}\right|\leq \frac{\Delta \omega }{2}}CWT_{x}\left(a_{k},b\right){a_{k}}^{-\frac{3}{2}}(\Delta a{)}_{k},$$

where $a_{k}-a_{k-1}=(\Delta a{)}_{k}$ is a discrete scale interval, and $\Delta \omega =\omega_{l}-\omega_{l-1}$ is a frequency interval. Finally, the wavelet coefficients after squeezing processing are converted into a time–frequency diagram. Taking the partial derivative of the wavelet coefficients (with respect to *b*) in the continuous wavelet transform, $CWT_{x}(a,b)$ yields an instantaneous frequency *ω_x_* (*a*, *b*), given by the following [34]:

(4) $$\omega_{x}\left(a,b\right)=\left\{\begin{array}{l} -i(CWT_{x}(a,b){)}^{-1}\frac{\partial }{\partial b}CWT_{x}(a,b),\left|CWT_{x}\left(a,b\right)>0\right|\\ \infty ,\left|CWT_{x}\left(a,b\right)=0\right| \end{array}\right..$$

This expression maps the wavelet coefficients $CWT_{x}(a,b)$ from the time–scale plane to the time–frequency plane, thereby obtaining a time–frequency diagram of the original rolling bearing vibration time-domain signal. The resulting time–frequency diagrams offer higher sparsity, time–frequency resolution, and aggregation potential.

### 2.3. Construction of the Transfer Residual Convolutional Neural Network (TRCNN)

In this study, TRCNN was used to automatically learn the time–frequency diagram features and achieve classification diagnosis. The network contains two primary modules, namely a residual convolutional neural network (RCNN) and a transfer learning module.

**Figure 2:**
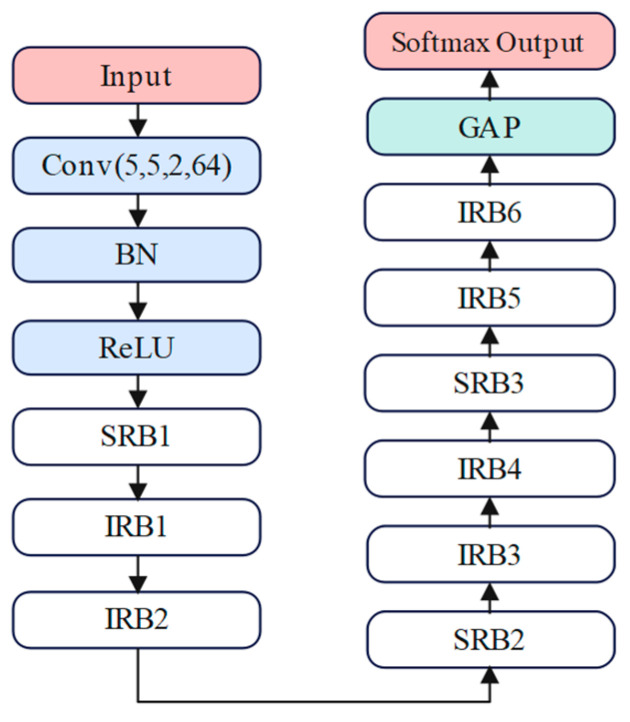
The deep residual network architecture. Conv (5, 5, 2, 64) represents a convolutional layer, BN represents a batch normalization layer, ReLU represents an activation function layer, SRB1–3 and IRB1–6 represent residual blocks, GAP represents a global average pooling layer, and Softmax Output represents a fully connected output layer.

**Figure 3:**
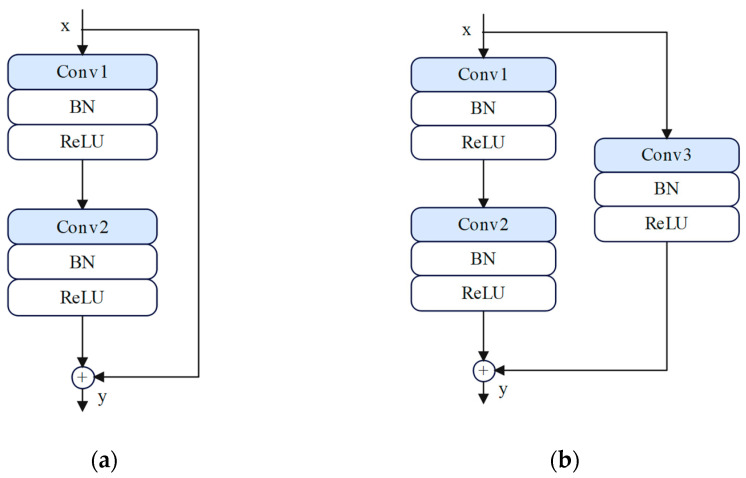
The structures of the (**a**) identity residual blocks and (**b**) downsampled residual blocks. Conv1–3 represent convolutional layers, BN represents a batch normalization layer, and ReLU represents an activation function layer.

The RCNN module, shown in Figure 2, accepts time–frequency diagrams as input and consists of a convolutional layer, a batch normalization layer, a ReLU activation function, a residual block, a global average pooling layer, and a fully connected output layer. The residual network uses two different residual block structures to facilitate smooth gradient transfer and the efficient updating of weights and biases in the deep CNN [36], as shown in Figure 3. The first is an identity residual block (IRB), shown in Figure 3a, which consists of two convolutional layers, 3 × 3 in size. An identity shortcut connected branch is also included, with both convolutional layers having a step size of 1 and a padding of 1. The second is a downsampled residual block (DRB), shown in Figure 3b, which consists of three convolutional layers. The first two layers employ a 3 × 3 convolutional kernel with a padding of 1. The first convolutional layer exhibits a step size of 2, thereby halving the size of the input feature map and reducing the burden of model training. Since the output and input can only be added together when the tensor dimensions are equal, a convolutional layer with a step size of 2 was added to the shortcut connection branch, which uses a 1×1 convolutional kernel with a padding of 0 [37].

**Figure 4:**
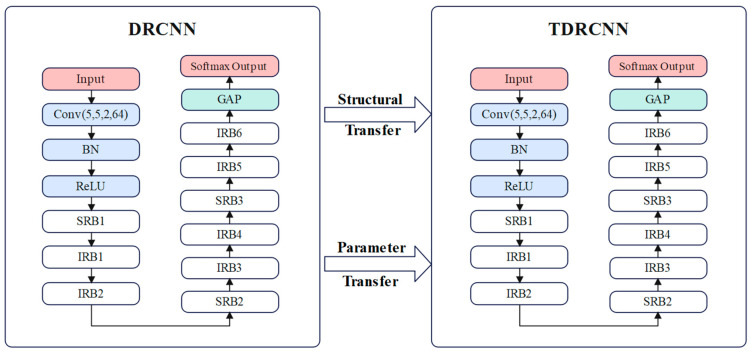
A schematic diagram of the transfer learning strategy. Conv (5, 5, 2, 64) represents a convolutional layer, BN represents a batch normalization layer, ReLU represents an activation function layer, SRB1–3 and IRB1–6 represent residual blocks, GAP represents a global average pooling layer, and Softmax Output represents a fully connected output layer.

The transfer learning module utilizes a pre-trained RCNN model, transferring its structure and parameters to the target task for improved learning efficiency with limited data. The model is restructured to align with the target domain’s data characteristics, leveraging knowledge from various conditions to enhance fault diagnosis accuracy. This transfer learning strategy, used to build the TRCNN, is illustrated in Figure 4.

The TRCNN model, built on two primary modules, uses time–frequency diagrams from the SWT as input. High-level features are extracted through the nonlinear transformation layer, processed via a global average pooling layer, and sent to the fully connected output layer. Transfer learning enables the effective classification of the time–frequency diagrams with limited samples, achieving state recognition and fault classification of rolling bearing signals.

## 3. Experimental Validation

### 3.1. Experimental Settings and Data Sources

**Figure 5:**
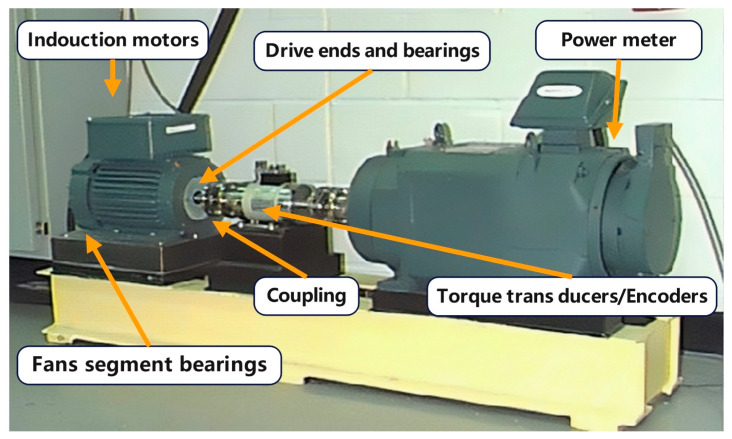
An illustration of the experimental setup.

This study utilized the vibration signal dataset developed by Case Western Reserve University (CWRU) [38]. The experimental setup, shown in Figure 5, included selecting a vibration signal at the drive end, with a frequency of 12 kHz. The experimental data exhibited 12 different states, including one normal state and three types of faults (i.e., inner ring, rolling body, and outer ring), each of which exhibited four different fault sizes (i.e., 0.007, 0.014, 0.021, and 0.028 inches). A lack of available fault signals in the dataset for the outer ring sizes of 0.028 inches limited the total number of states to 12. In each state, the data were segmented using a non-overlapping time window with 1024 points, from which 110 samples were selected to form the study dataset. The samples were then categorized into four sub-datasets, *a*, *b*, *c*, and *d*, corresponding to the four loads (i.e., 0, 1, 2, and 3 horsepower), as detailed in Table 1.

The experiments were conducted on a 3090 server, with MATLAB 2022a used for the time–frequency analysis of vibration signals and the construction of time–frequency diagrams. The TRCNN model was implemented in the Python 3.8 environment using the Pytorch framework.

### 3.2. Analysis of TRCNN Diagnostic Results

**Figure 6:**
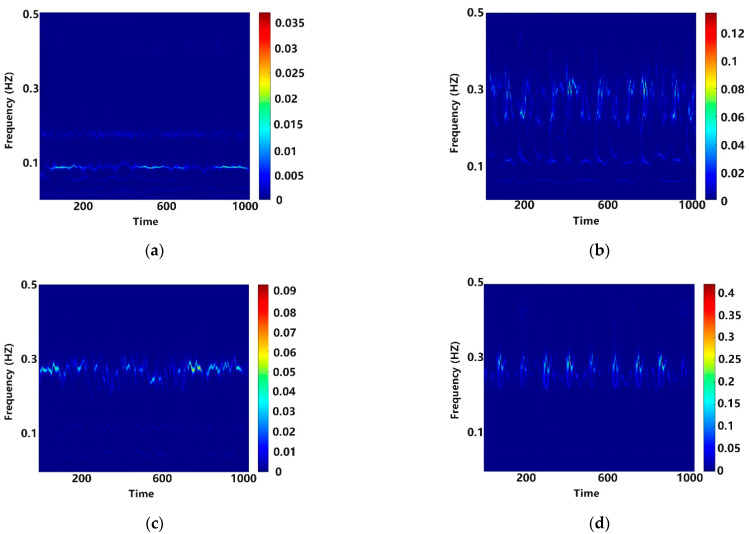
Time–frequency diagrams from various states, including the (**a**) normal state, (**b**) 0.007-inch inner ring fault, (**c**) 0.007-inch rolling body fault, and (**d**) 0.007-inch outer ring fault time–frequency diagrams.

Vibration signals from the dataset, segmented in Section 3.1, were first converted to time–frequency diagrams using a simultaneous SWT for each section of data during subsequent fault diagnosis by the TRCNN. Figure 6 shows the time–frequency diagrams of four different states, including the normal state under zero load, a 0.007-inch inner-ring fault, a 0.007-inch rolling-body fault, and a 0.007-inch outer-ring fault. The generated time–frequency diagrams clearly demonstrate the characteristics of these signals in both the time and frequency domains, which was very helpful for identifying the type of bearing fault.

The TRCNN was then used to classify the time–frequency diagrams and complete the fault diagnosis process. In this paper, fault diagnosis accuracy was used as an algorithm evaluation metric, as the bearing dataset in different working conditions was included to validate the proposed method through simulation experiments. The ratio of the training set to the validation set samples was 8:2, and the AdamW optimizer was employed in the setting of TRCNN parameters by considering both computational efficiency and memory usage. The weight decay was set to 1 × 10^−4^ to minimize the loss function and improve the training speed. Detailed parameter settings are shown in Table 2, with parameters for the convolutional layer of the residual block provided in Table 3.

**Figure 7:**
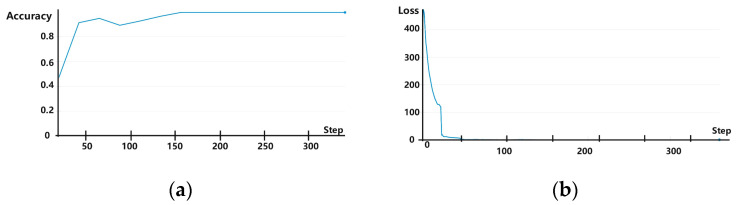
(**a**) Accuracy and (**b**) loss curves for experiments involving one-time transfer learning.

**Figure 8:**
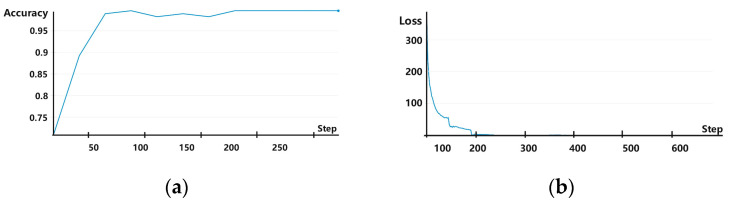
(**a**) Accuracy and (**b**) loss curves for experiments involving two-time transfer learning.

**Figure 9:**
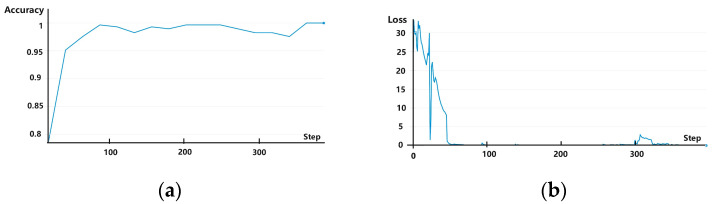
(**a**) Accuracy and (**b**) loss curves for experiments involving three-time transfer learning.

The experiments were conducted using one-time (i.e., from dataset A → B), two-time (i.e., from dataset A → B and then from B → C), and three-time (i.e., from dataset A → B, B → C, and finally, C → D) transfer learning. In each scenario, samples from the training set were iterated for 50 rounds, and data in the validation set were used for verification. Experimental results showed that the rolling bearing fault diagnosis accuracy reached 99.65% for one-time, 99.65% for two-time, and 100% (i.e., all fault diagnoses were correct) for three-time transfer learning. The accuracy and loss curves are shown for one-, two-, and three-time transfers in Figure 7, Figure 8 and Figure 9, respectively, with the horizontal coordinate indicating the number of training iterations and the vertical coordinates indicating the diagnostic accuracy and model training loss. It is evident from these figures that when using transfer learning, both the accuracy and loss curves exhibited stable convergence after sufficient iterations, indicating that the model did not experience overfitting. Thus, the number of training samples used in these experiments was sufficient to support the employed multilayer network structure, ensuring the reliability of fault diagnosis accuracy.

## 4. Comparative Tests

Based on the proposed method and the components of the model, this section separately verifies the superiority of SWT in time–frequency analysis methods, the superiority of residual networks in TRCNN, the superiority of transfer learning in TRCNN, and the superiority of the proposed method based on the CWRU dataset. The resulting fault diagnostic accuracy in each case is shown in Table 4.

**Figure 10:**
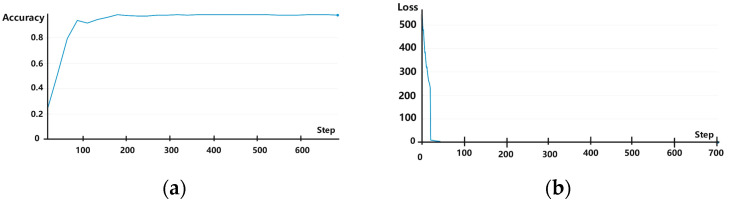
(**a**) Accuracy and (**b**) loss curves for experiments without transfer learning.

**Figure 11:**
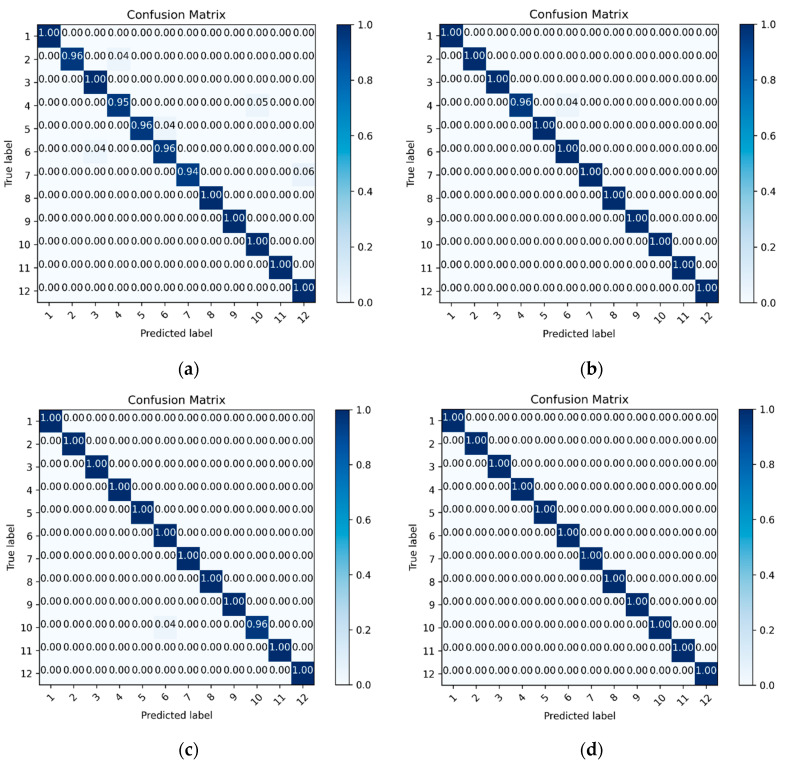
Fault diagnosis confusion matrices for four transfer scenarios, including (**a**) without, (**b**) one-time, (**c**) two-time, and (**d**) three-time transfer learning.

To verify the superiority of transfer learning in the TRCNN model, we construct a residual convolutional neural network model (RCNN), without transfer learning. The same experiments were then performed to produce the accuracy and loss curves shown in Figure 10. The experimental results showed that rolling bearing fault diagnosis accuracy reached 98.26%, which was lower than the fault diagnosis accuracy when performing one-, two-, and three-time transfer learning. The average accuracy of all methods utilizing transfer learning was much higher than that of the algorithm trained from scratch using a small number of target samples, indicating that transfer learning significantly improved the classification accuracy of deep networks in cases of small sample quantities and variable working conditions. To provide a more intuitive comparison, fault diagnosis confusion matrices were generated for cases of no transfer, as well as one-, two-, and three-time transfer, as shown in Figure 11. It is evident from these confusion matrices that the feature distributions learned by RCNN exhibited more aliasing in class 2 and class 4, class 4 and class 10, class 5 and class 6, class 3 and class 6, and class 7 and class 12, while only one sample class displayed aliasing for one-time and two-time transfers, and no sample aliasing occurred for the three-time transfers in the feature distributions learned by TRCNN. The resulting fault diagnosis accuracy reached 100% when using three-time transfer learning, with better inter-class separability and higher intra-class aggregation, confirming the effectiveness of the transfer learning step introduced in this paper.

To verify the superiority of the residual network in the TRCNN model and the superiority of SWT in the time–frequency analysis methods, we compared the model without the residual network (TCNN) with the model containing the residual network (TRCNN). Additionally, different time–frequency analysis methods were employed, and the fault diagnosis accuracy was obtained using the A → D transfer learning dataset, as shown in Table 5 [37].

As shown in Table 5, the fault diagnosis accuracy obtained using SWT for time–frequency processing is the highest. Moreover, although the fault diagnosis accuracy of TCNN and TRCNN varies across different time–frequency analysis methods, TRCNN consistently achieves better test results than does TCNN. This indicates that the residual structure is more advantageous than the traditional stacked structure.

**Figure 12:**
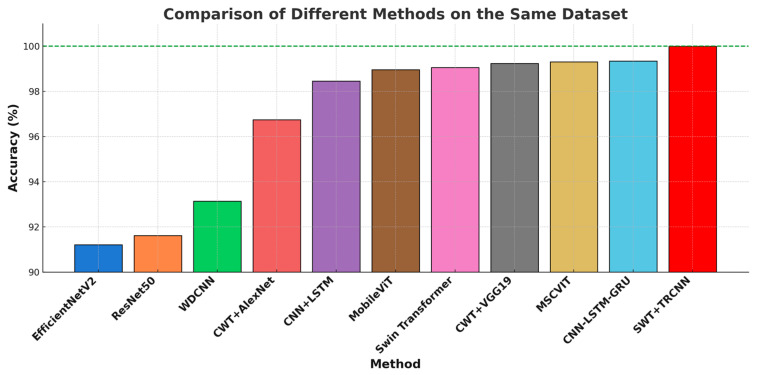
Experimental comparisons of different methods applied to the same dataset.

To verify the superiority of the method proposed in this paper using the CWRU dataset, we compared it with other methods based on CWRU that are similar in principle to those used in this study and currently advanced in the industry. These include the CNN-LSTM combination technique [39], a multi-scale convolutional block attention module—a vision transformer (MSCVIT) bearing fault diagnosis model [40], a CNN-LSTM-GRU Model [41], and CWT combined with either the AlexNet or VGG19 networks [42]. Figure 12 shows that, measured by fault diagnosis accuracy, the proposed SWT and TRCNN combination method outperforms all other techniques.

## 5. Conclusions

A novel technique, combining the theories of SWT, transfer learning, and residual learning, was introduced for rolling bearing fault diagnosis. This method takes the time–frequency diagrams constructed by SWT as input for a TRCNN, which is then applied to feature learning, fault classification, and diagnosis. The introduction of compression processing makes the wavelet coefficients more concentrated at the estimated instantaneous frequency so that the energy can be more fully gathered on the actual corresponding frequency components and solves the difficulty of feature extraction caused by the non-stationarity of fault signals. The introduction of identity residual blocks, downsampling residual blocks, and transfer learning addresses the challenge of low fault diagnosis accuracy caused by small sample quantities. The CWRU dataset was used to perform one-time, two-time, and three-time transfer learning under different working conditions, with diagnostic accuracy reaching 99.65% for one-time and two-time transfers and 100% for three-time transfers. Comparative experiments showed that the proposed method improved fault diagnosis accuracy by 1.74% over RCNN, 1.28% over TCNN, 1.62% over STFT, 1.73% over WT, 2.83% over PWVD, and 1.39% over STFA-PD. These results provide a meaningful foundation for future developments in the field of fault diagnosis.

## Figures and Tables

**Table 1:** The bearing dataset after classification and data segmentation.

Fault Type	Size (Inches)	Load	Sample Size	Label
Normalcy	0	0/1/2/3	110/110/110/110	1
Inner Ring Failure	0.007	0/1/2/3	110/110/110/110	2
	0.014	0/1/2/3	110/110/110/110	3
	0.021	0/1/2/3	110/110/110/110	4
	0.028	0/1/2/3	110/110/110/110	5
Rolling Body Failure	0.007	0/1/2/3	110/110/110/110	6
	0.014	0/1/2/3	110/110/110/110	7
	0.021	0/1/2/3	110/110/110/110	8
	0.028	0/1/2/3	110/110/110/110	9
Outer Ring Failure	0.007	0/1/2/3	110/110/110/110	10
	0.014	0/1/2/3	110/110/110/110	11
	0.021	0/1/2/3	110/110/110/110	12

**Table 2:** Network parameter settings.

Parameters	Pre-Training Phase	Transfer Learning Phase
Optimization Algorithm	AdamW	AdamW
Weight Decay	1 × 10^−4^	1 × 10^−4^
Learning Rate	0.01	0.01
Batch Size	48	48
Epochs	50	50

**Table 3:** Residual block parameter details.

Residual Block	Convolutional Layer Parameters		
	Conv1	Conv2	Conv3
SRB1	Conv (3, 3, 1, 64)	Conv (3, 3, 2, 64)	Conv (1, 1, 2, 64)
IBR1	Conv (3, 3, 1, 64)	Conv (3, 3, 1, 64)	\
IBR2	Conv (3, 3, 1, 64)	Conv (3, 3, 1, 64)	\
SBR2	Conv (3, 3, 1, 128)	Conv (3, 3, 2, 128)	Conv (1, 1, 2, 128)
IBR3	Conv (3, 3, 1, 128)	Conv (3, 3, 1, 128)	\
IBR4	Conv (3, 3, 1, 128)	Conv (3, 3, 1, 128)	\
SBR3	Conv (3, 3, 1, 256)	Conv (3, 3, 2, 256)	Conv (1, 1, 2, 256)
IBR5	Conv (3, 3, 1, 256)	Conv (3, 3, 1, 256)	\
IBR6	Conv (3, 3, 1, 256)	Conv (3, 3, 1, 256)	\

**Table 4:** A comparison of the method proposed in this paper and conventional techniques.

Comparison	Method	Diagnostic Accuracy (%)
Comparison of Transfer Learning	No Transfer	98.26
	One-Time Transfer A → B	99.65
	Two-Time Transfer A → B → C	99.65
	Three-Time Transfer A → B → C → D (Proposed Method)	**100**
Comparison of Time–Frequency Analysis	STFT + TRCNN	98.38
	WT + TRCNN PWVD + TRCNN STFA-PD + TRCNN SWT + TRCNN (Proposed Method)	98.27 97.17 98.61 **100**
Comparison of Residual Networks	SWT + TCNN SWT + TRCNN (Proposed Method)	98.72 **100**
Comparison of Different Methods Applied to the Same Dataset	EfficientNetV2	91.21
	ResNet50	91.61
	WDCNN	93.14
	CWT + AlexNet	96.74
	CNN + LSTM	98.46
	MobileViT Swin Transformer CWT + VGG19 MSCVIT CNN-LSTM-GRU SWT + TRCNN (Proposed Method)	98.96 99.06 99.24 99.31 99.34 **100**

STFT, short-time Fourier transform; WT, wavelet transform; PWVD, pseudo Wigner–Ville distribution; STFA-PD, first-order primal-dual algorithm based sparse time–frequency analysis method; TCNN, transfer convolutional neural network; WDCNN, deep convolutional neural networks with wide first-layer kernel; CWT, continuous wavelet transform; CNN, convolutional neural network; LSTM, long short-term memory; MSCVIT, multi-scale convolutional block attention module—a vision transformer; GRU, gated recurrent unit.

**Table 5:** Fault diagnosis accuracy of different methods.

	STFT	WT	PWVD	STFA-PD	SWT
TRCNN	98.38%	98.27%	97.17%	98.61%	**100%**
TCNN	97.58%	96.64%	93.06%	95.87%	98.72%

## Data Availability

The CWRU dataset is available at https://engineering.case.edu/bearingdatacenter (accessed on 23 August 2024).
